# Beyond the Visual Acuity: Assessing the Visual Function in mCNV Patients After Anti-VEGF Treatment

**DOI:** 10.3389/fmed.2021.709584

**Published:** 2021-08-31

**Authors:** Songshan Li, Limei Sun, Xiujuan Zhao, Zhaotian Zhang, Xiaoling Luo, Xiaoyan Ding

**Affiliations:** State Key Laboratory of Ophthalmology, Retina Division, Zhongshan Ophthalmic Center, Sun Yat-sen University, Guangzhou, China

**Keywords:** myopic choroidal neovascularization, visual function, metamorphopsia, microperimetry, mCNV

## Abstract

**Purpose:** To investigate visual function and vision-related quality of life (VR-QoL) changes in patients with myopic choroidal neovascularization (mCNV) after ranibizumab treatment.

**Methods:** Quantitatively evaluate the objective tests of visual function (visual acuity, microperimetry, and metamorphopsia by m-Charts) before and after 3+prn (pro re neta) ranibizumab treatment for 1 year. The National Eye Institute 25-Item Visual Function Questionnaire (VFQ-25) was performed to evaluate the VR-QoL.

**Results:** A total of 57 eyes of 57 patients were included in this study. The median average metamorphopsia score was 0.65 before treatment and improved to 0.45 after treatment (*p* = 0.0003). There was also a significant difference in the average threshold, macular integrity, and proportion of patients with stable fixation by the microperimetry (*p* < 0.000, *p* < 0.0001, and *p* = 0.03, respectively). After treatment, the VR-QoL composite, general vision subscale, and vision-related mental health subscale score were increased with borderline or statistical significance (*p* = 0.088, *p* = 0.0038, and *p* = 0.012, respectively). Subgroup analysis demonstrated parallel improvement of the VR-QoL score, metamorphopsia, average macular threshold, and fixation stability in patients with or without visual acuity increase. By multiple linear regression analysis, the VFQ-25 score after anti-VEGF treatment was only associated with the baseline VFQ-25 score and macular integrity. Improvements in the VFQ-25 score were only associated with changes in the metamorphopsia score.

**Conclusions:** Integral lifting in several aspects of visual function was observed in mCNV after ranibizumab treatment. Macular integrity and metamorphopsia, but not visual acuity, were associated with VR-QoL.

## Introduction

Myopic choroidal neovascularization (mCNV) is one of the most common vision-threatening complications of pathological myopia, affecting 5–11% of patients with pathological myopia and 0.04–0.05% of the general population (1). It is particularly prevalent among young and middle-aged Asians (2). Vision-related quality of life (VR-QoL) is significantly compromised in mCNV patients (3, 4), probably caused by not only the decrease in visual acuity (VA) but also the presence of metamorphopsia, scotomata, and fixation ability (5, 6). To date, VA is the standard, most common way to evaluate visual function in mCNV. In most clinical studies, it is even the only way to assess the visual function (7, 8). VR-QoL and other aspects of visual function, including the fixation ability, metamorphopsia, and scotomata, are barely studied.

To date, several quantifiable measurements have been developed for a wide recognition of the total visual ability and subjective perception of VR-QoL, including microperimetry, contrast sensitivity, metamorphopsia, and the 25-item National Eye Institute Visual Function Questionnaire (VFQ-25). The macular sensitivity measured by microperimetry has been used for the assessment of macular function in several diseases and showed correlation with the VA or VFQ-25 in several diseases (9, 10). Some studies in wet age-related macular degeneration (wAMD) demonstrated the discrepancy between VA and other visual functions. Tran et al. reported severe impairment of macular sensitivity by microperimetry in AMD patients with good central visual acuity (11). Discrepant changes of VA and metamorphopsia improvement were also reported after treatment in wAMD or after surgery in patients with macular holes (12, 13).

In patients with mCNV, VA improved about 13–15 Early Treatment Diabetic Retinopathy Study (ETDRS) letters 1 year after a scheduled anti-VEGF treatment (7, 8, 14). Without treatment, VA may drop to 20/200 or worse within 5 years in patients with mCNV (15, 16). However, very few studies paid attention to changes in other aspects of visual function impairment except VA. In the limited studies, the improvement of macular sensitivity or fixation ability was noticed after bevacizumab or photodynamic therapy but failed to correlate the improvement with patient-reported outcomes (5, 17, 18). It was observed in the MYRROR study (VEGF trap-eye in choroidal neovascularization secondary to pathologic myopia, NCT01249664) that more than 10% mCNV patients did not achieve substantial VA improvement (≥5 letters) after 12 months of anti-VEGF treatment (7), while the changes in VR-QoL and other aspects of visual function in these patients remain unknown.

During anti-VEGF treatment, monitoring visual function and VR-QoL not limited to the VA could be helpful for recognizing the entire picture of the disease and for assessing the benefits of anti-VEGF therapy. Therefore, the purpose of this study was to investigate visual functional improvements and VR-QoL changes during anti-VEGF treatment in patients with mCNV. Indicators in addition to VA were prospected for visual function assessment.

## Methods

### Patients

This retrospective case series was conducted in Zhongshan Ophthalmic Center, Sun Yat-Sen University with the permission of the Institutional Review Board (2015MEKY053). All investigations followed the tenets of the Declaration of Helsinki. A total of 57 eyes of 57 patients with active unilateral mCNV were enrolled in this study from March 2014 to July 2018, including 20 patients who participated in a previous published study (SMILE: a single blind clinical trial “Treatment and assessment Strategy for MyopIc CNV with LucEntis: a single-center, prospective randomized controlled study,” NCT03042871) (14). The inclusion criteria were as follows: (1) unilateral active subfoveal or juxtafoveal CNV associated with high myopia (spherical equivalence < −6.0 D or axial length > 26 mm) confirmed by fundus fluorescein angiography (FFA) with a hyperfluorescent CNV network on early frames and leakage on late frames; (2) patients with baseline best-corrected visual acuity (BCVA) in the affected eye from 24 to 73 ETDRS letters; and (3) patients who received 3+prn (pro re neta) intravitreal ranibizumab treatments for 12 months. The exclusion criteria were as follows: (1) presence of other ocular diseases or evidence of any condition other than CNV associated with high myopia that affected the VA for both eyes (including moderate to dense lens opacity); (2) any anti-VEGF therapy performed within the last 6 months; (3) previous photodynamic therapy (PDT); (4) intraocular surgery performed within the last 3 months; and (5) pregnancy or severe systemic conditions, including uncontrolled systemic hypertension, or any history of thromboembolic or ischemic cardiovascular diseases. The retreatment was administered in patients who met any of the following criteria: (a) reduction of BCVA > 5 letters from the previous visit; (b) increase in central retinal thickness (CRT) > 50 μm from the previous visit; new or persistent cystic retinal changes, subretinal fluid or pigment epithelial detachment; and (c) new or persistent bleeding or leakage in FFA or fundus examination.

BCVA, optical coherence tomography (OCT), and FFA were measured before and after 12-month ranibizumab treatments. Quantitative evaluation of metamorphopsia using m-Charts (Inami Co., Tokyo, Japan) and macular function assessed by microperimetry (MAIA, Centervue, Italy) were performed on each patient. The m-Charts were used to analyze the metamorphopsia score by quantifying the minimum visual angle (from 0 to 2 degrees) of a dotted straight line for patients to recognize the distortion. The measurement was conducted in a bright light at a distance of 30 cm. Both vertical and horizontal metamorphopsia scores were measured and repeated three times. The mean m-Charts value was used in the statistical analysis. Microperimetry was performed using a macular integrity assessment (MAIA) by an expert 4-2 examination covering 10 degrees of diameter of the macular area. The examination included 37 measurement points in three circles with 2, 6, and 10 degrees of diameter, respectively. The stimulus was Goldman III in magnitude and lasted 200 ms. The illumination of the stimulus was distributed from 0 to 36 dB. The results of average threshold of macular sensitivity, macular integrity index, P1, P2, 63% bivariate contour ellipse area (BCEA), and 95% BCEA were analyzed and classified. Fixation stability was classified according to Fujii et al. (19): (1) If P1 is ≥75%, the fixation was classified as “stable”; (2) if P1 < 75% but P2 is ≥75%, the fixation was classified as “relatively unstable”; and (3) if P2 < 75%, the fixation was classified as “unstable.” VR-QoL, using the National Eye Institute 25-Item Visual Function Questionnaire (VFQ-25), was reported in all patients before and 12 months after 3+prn intravitreal ranibizumab treatment. The composite score and the score of each subscale were calculated followed the specifications of the National Eye Institute (20).

### Statistical Analysis

Statistical analysis was performed using GraphPad (GraphPad Software, CA, USA) or SPSS (SPSS Inc., Chicago, IL, USA). Shapiro–Wilk test was used to test for the normality distribution. Comparisons of the continuous variables were performed using the two-tailed Student's *t*-test or the Mann–Whitney test when appropriate. Paired two-tailed Student's *t*-tests or Wilcoxon matched-pairs signed rank tests were used for paired data. Analysis of the dichotomous variables was performed using the chi-square test (or Fisher's exact test when appropriate). To determine the factors that might be correlated with the VA and the VR-QoL score, multiple linear regression analyses by the stepwise methods were conducted. Three dependent variables were used: the final BCVA after 12 months of treatment, the final composite VR-QoL score, and the improvement of the composite VR-QoL score. According to the reported possible prognostic factors, the independent variables included gender, age, BCVA, macular integrity, average threshold, P1, P2, 63% BCEA area, 95% BCEA area, composite VR-QoL score, and average metamorphopsia score (3, 9, 10, 20). The level of significance was set at *p* < 0.05.

## Results

### Demographics

A total of 57 eyes in 57 patients were enrolled in this study. Overall, 33/57 (57.9%) of the patients were female, and the mean age was 50.51 ± 13.80 years old (range: 20–76 years). The average spherical equivalent refractive error was −11.76 ± 5.03 D, and the mean axial length was 28.65 ± 1.40 mm. The average baseline BCVA was 51.89 ± 14.89 ETDRS letters. mCNV was located subfoveally in 82.5% (47/57) patients. The other 17.5% (10/57) was juxtafoveal CNV (Table 1).

**Table 1:** Demographic and baseline characters.

**Characteristics**	**Mean ± SD**	**Median of total**	**Min of total**	**Max of total**
*n*	57			
Male/female	24/33			
Age(year)	50.51 ± 13.80	53	20	76
BCVA (ETDRS)	51.89 ± 14.89	52	24	73
Axial length (mm)	28.65 ± 1.40	28.77	26.16	32.01
Spherical equivalent	−11.76 ± 5.03	−10.75	−21.25	−6.25
CNV location (subfoveal/juxtafoveal)	47/10			

### VA and Structural Improvement in Patients With mCNV Before and After Treatment

After 12-month 3+prn intravitreal ranibizumab (IVR) treatments, the average BCVA increased from 51.89 ± 14.89 to 62.95 ± 14.52 letters (*p* < 0.0001). Of these, more than 5-letter improvements were found in 66.7% (38/57) patients. The central retinal thickness (CRT) decreased from 261.6 ± 85.7 to 215.9 ± 70.1 μm (*p* < 0.0001) (Table 2).

**Table 2:** Functional visual performance before and after anti-VEGF treatment.

**Characteristics**	**Baseline**	**After treatment**	***P***
	**Mean ± SD (median)**	**Mean ± SD (median)**	
BCVA (ETDRS)	51.89 ± 14.89 (52)	62.95 ± 14.52 (66)	***<0.0001***
CRT (um)	261.6 ± 85.7 (257.5)	215.9 ± 70.1 (210.5)	***<0.0001***
Average metamorphopsia score	0.90 ± 0.70 (0.65)	0.58 ± 0.55 (0.45)	0.0003^*^
Macular integrity	94.65 ± 17.03 (100)	82.81 ± 25.43 (97.6)	<0.0001^*^
Average threshold	18.50 ± 5.99 (19.3)	22.42 ± 5.11 (23.6)	***<0.0001***
P1	45.14 ± 30.17 (43)	63.25 ± 28.58 (70)	<0.0001^*^
P2	72.60 ± 26.27 (84)	84.32 ± 28.58 (91)	0.0006^*^
Unstable/Relatively unstable/Stable	21 (36.8%)/23 (40.4%)/13 (22.8%)	14 (24.6%)/18 (31.6%)/25 (43.9%)	***0.03**^**#**^*
Area of 63% BCEA (mm^2^)	12.74 ± 13.13 (7.35)	6.92 ± 7.46 (3.90)	0.0003^*^
Area of 95% BCEA (mm^2^)	38.17 ± 39.36 (22.15)	21.31 ± 24.40 (11.60)	0.0007^*^

* *Wilcoxon matched-pairs signed rank test*.

# *Chi-square test for trend*.

### Functional Visual Performance in Patients With mCNV Before and After Treatment

The functional visual changes following IVR treatments are summarized in Table 2. The mean average metamorphopsia score was 0.90 ± 0.70 (median 0.65) before treatment and improved to 0.58 ± 0.55 (median 0.45) after treatment (*p* = 0.0003). There was also a significant difference in the average threshold (18.50 ± 5.99 median 19.3 to 22.42 ± 5.11 median 23.6, *p* < 0.0001) and macular integrity (94.65 ± 17.03 median 100 to 82.81 ± 25.43 median 97.6, *p* < 0.0001) by the microperimetry. Fixation was significantly more stable in P1, P2, 63% area of BCEA, and 95% area of BCEA after treatment, when compared with those at baseline (details in Table 2). The proportion of patients with normal fixation stability also increased from 22.8% (13/57) to 43.9% (25/57) (*p* = 0.03).

### Changes of VR-QoL in Patients With mCNV Before and After Treatment

The changes of VR-QoL measured using the VFQ-25 questionnaire are summarized in Table 3. After IVR treatment, most subscale scores and the composite score were numerically increased, except for the “peripheral vision subscale.” Although no statistical significance was observed (*p* = 0.088), the composite score modestly increased from 65.42 ± 14.05 to 68.12 ± 14.28. The “general vision subscale” and the “vision related mental health subscale” significantly increased after treatment (26.93 ± 17.21 to 35.36 ± 17.47, *p* = 0.0038 and 47.48 ± 19.97 to 53.24 ± 21.09, *p* = 0.012, respectively).

**Table 3:** Subjective QoL score by VFQ25 before and after anti-VEGF treatment.

**Subscales**	**Baseline**	**After**	***P***
General health	40.35 ± 19.91	44.02 ± 17.63	0.58
General vision	26.93 ± 17.21	35.36 ± 17.47	***0.0038***
Ocular pain	72.37 ± 17.56	74.33 ± 16.59	0.45
Near activities	66.52 ± 23.01	71.27 ± 24.42	0.11
Distance activities	71.42 ± 21.02	74.25 ± 18.10	0.21
Vision related social functioning	86.18 ± 19.86	86.38 ± 16.22	0.84
Vision related mental health	47.48 ± 19.97	53.24 ± 21.09	***0.012***
Vision related role difficulties	52.63 ± 25.52	53.79 ± 22.73	0.84
Vision related dependency	59.94 ± 24.11	62.80 ± 25.22	0.43
Driving	62.30 ± 39.58	66.67 ± 39.09	0.39
Color vision	91.07 ± 16.81	92.41 ± 15.01	0.81
Peripheral vision	82.14 ± 21.17	78.57 ± 21.55	0.10
Composite score	65.42 ± 14.05	68.12 ± 14.28	0.088

### Parallel Improvement of VR-QoL in Subgroups With/Without VA Improvement

As mentioned above, not all patients gained significant VA improvement after the treatment. Among the 57 patients, two-thirds (38/57, 66.7%) showed a BCVA improvement of more than 5 ETDRS letters (mean ± SD, 15.82 ± 7.81 letters) and were subdivided into group A, and the other one-third (19/57, 33.3%) of patients were subdivided into group B [with BCVA improvement ≤ 5 ETDRS letters (mean ± SD, 1.53 ± 3.75 letters)]. No patients experienced a VA loss of more than 5 ETDRS letters. The average injection number was 3.31 ± 0.69 in group A, while it was 3.61 ± 0.91 in group B (*p* = 0.17). The median age was 53.5 years old (range: 23–76 years) in group A and 50 years old (range: 20–71 years) in group B (*p* = 0.42). In addition, no statistical difference was found about the sex between the two subgroups (male/female, 14/24 in group A and 10/9 in group B, *p* = 0.27).

The changes of VR-QoL before and after treatment between the two subgroups were then analyzed (Table 4). The composite score increased from 65.33 ± 13.14 to 68.44 ± 14.00, and the score of vision subscale statistically increased from 27.76 ± 17.89 to 36.22 ± 17.54 (*p* = 0.003) in group A. Despite poor improvement of VA, the patients in group B gained parallel improvement in VR-QoL compared with group A. The composite score increased from 65.60 ± 16.09 to 67.48 ± 15.19, and the score of vision subscale increased from 25.26 ± 16.11 to 33.68 ± 17.71 (*p* = 0.011). Furthermore, the change of vision subscale before and after treatment was comparable between the two subgroups (*p* = 0.976, Table 5).

**Table 4:** Visual function and VR-QoL parameters in BCVA improved subgroup and BCVA sustained subgroup.

**Parameters**	**BCVA changes ≤ 5 letters**	**BCVA changes > 5 letters**	***P***
	***n* = 19**	***n* = 38**	
Baseline BCVA	55.58 ± 16.13 (52)	50.05 ± 14.08 (48.5)	0.189
Final BCVA	57.11 ± 16.15 (59)	65.87 ± 12.88 (69)	***0.03***
*P* (baseline vs. final)	0.093	***<0.0001***	
Baseline Macular integrity	88.22 ± 26.96 (100)	97.86 ± 7.33 (100)	0.139^*^
Final integrity	84.54 ± 29.10 (100)	81.95 ± 23.76 (96.15)	0.229^*^
*P* (baseline vs. final)	0.285^*^	<0.0001^*^	
Baseline average threshold	18.97 ± 6.99 (19.8)	18.27 ± 5.51 (19.1)	0.683
Final average threshold	21.93 ± 5.57 (22.5)	22.66 ± 4.93 (23.85)	0.614
*P* (baseline vs. final)	***0.001***	***<0.0001***	
Baseline P1	48.74 ± 31.51 (46)	43.34 ± 29.73 (41)	0.559^*^
Final P1	68.00 ± 31.12 (86)	60.87 ± 27.34 (62.00)	0.334^*^
*P* (baseline vs. final)	0.005^*^	0.004^*^	
Baseline P2	74.16 ± 27.97 (84)	71.82 ± 25.73 (83.5)	0.703^*^
Final P2	86.79 ± 16.93 (96.00)	83.08 ± 17.87 (86.00)	0.278^*^
*P* (baseline vs. final)	0.010^*^	0.017^*^	
Baseline 63%BCEA area	14.58 ± 17.85 (6.65)	11.96 ± 10.77 (7.80)	0.955^*^
Final 63%BCEA area	5.95 ± 7.59 (2.3)	7.41 ± 7.45 (6.2)	0.184^*^
*P* (baseline vs. final)	0.009^*^	0.012^*^	
Baseline 95%BCEA area	43.68 ± 53.47 (19.95)	35.86 ± 32.28 (23.40)	0.947^*^
Final 95%BCEA area	17.78 ± 22.71 (6.8)	23.08 ± 25.30 (17.60)	0.190^*^
*P* (baseline vs. final)	0.010^*^	0.024^*^	
Baseline metamorphopsia score	0.91 ± 0.71 (0.60)	0.89 ± 0.70 (0.70)	0.946^*^
Final metamorphopsia score	0.64 ± 0.64 (0.50)	0.55 ± 0.51 (0.425)	0.858^*^
*P* (baseline vs. final)	0.016^*^	0.010^*^	
Baseline composite score	65.60 ± 16.09 (70.45)	65.33 ± 13.14 (65.42)	0.946
Final composite score	67.48 ± 15.19 (65.79)	68.44 ± 14.00 (67.625)	0.813
*P* (baseline vs. final)	0.479	0.103	
Baseline vision subscale score	25.26 ± 16.11 (20)	27.76 ± 17.89 (20)	0.610
Final vision subscale score	33.68 ± 17.71 (40)	36.22 ± 17.54 (40)	0.612
*P* (baseline vs. final)	***0.011***	***0.003***	
Baseline mental health subscale score	46.38 ± 19.13 (43.75)	48.03 ± 20.61 (43.75)	0.772
Final mental health subscale score	51.64 ± 20.71 (56.25)	54.05 ± 21.51 (56.25)	0.689
*P* (baseline vs. final)	0.186	***0.034***	

* *Wilcoxon matched-pairs signed rank test or Mann–Whitney test*.

**Table 5:** Visual function and VR-QoL changes in BCVA improved subgroup and BCVA sustained subgroup.

	**BCVA change ≤ 5**	**BCVA change > 5**	***P***
*n*	19	38	
BCVA	1.53 ± 3.75 (1.0)	15.82 ± 7.81 (14.0)	***<0.0001***
Macular integrity	−3.68 ± 24.62 (−0.2)	−15.91 ± 24.44 (−2.55)	0.06^*^
Average threshold	2.96 ± 3.27 (2.1)	4.39 ± 4.25 (4.1)	0.81
P1	19.26 ± 25.53 (19.00)	17.53 ± 33.01 (14.50)	0.79^*^
P2	12.63 ± 20.75 (9.0)	11.26 ± 24.10 (3.0)	0.45^*^
63% BCEA area	−8.27 ± 13.56 (−3.45)	−4.55 ± 11.03 (−1.90)	0.46^*^
95% BCEA area	−24.80 ± 40.69 (−10.20)	−12.78 ± 33.13 (−5.00)	0.36^*^
Average metamorphopsia score	−0.27 ± 0.44 (−0.2)	−0.34 ± 0.68 (−0.18)	0.79^*^
General vision subscale	8.42 ± 15.37 (0.0)	8.24 ± 22.98 (0.0)	0.976
Mental health subscale	5.26 ± 16.70 (6.25)	5.57 ± 15.36 (6.25)	0.945
Composite score	1.88 ± 11.36 (2.42)	2.40 ± 8.73 (3.52)	0.521

* *Mann–Whitney test*.

### Parallel Resolution of Metamorphopsia in Subgroups With/Without VA Improvement

We then analyzed the metamorphopsia evaluation with or without BCVA improvement after ranibizumab treatment (Table 4). The average m-Charts score significantly improved from 0.89 ± 0.70 (median 0.70) to 0.55 ± 0.51 (median 0.425) (*p* = 0.010) in group A and from 0.91 ± 0.71 (median 0.60) to 0.64 ± 0.64 (median 0.50) in group B (*p* = 0.016). The resolution of metamorphopsia was also comparable in the two subgroups (*p* = 0.79).

### Visual Function by Microperimetry in Subgroups With/Without VA Improvement

**Figure 1:**
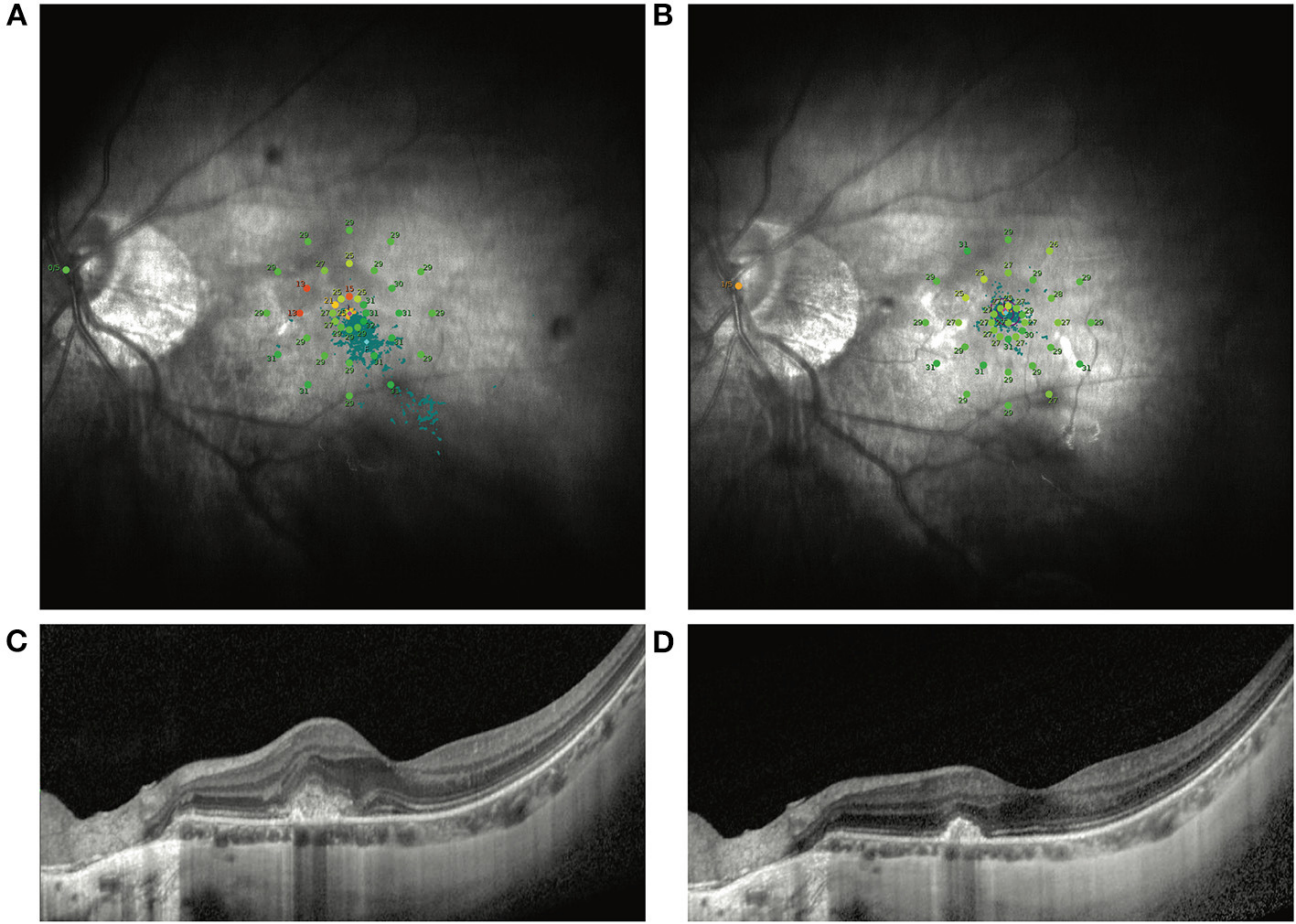
Representative MP1 findings in an mCNV patient without VA improvement after ranibizumab treatment. **(A)** Shows the image of the MP1 before ranibizumab treatment in an mCNV patient with baseline BCVA as 67 ETDRS letters. After treatment, the VA increased by only 1 ETDRS letter. Nevertheless, the macular sensitivity and fixation improved dramatically by MP1 **(B)**. OCT images also show the remission of the CNV lesion in the macular **(C,D)**.

**Figure 2:**
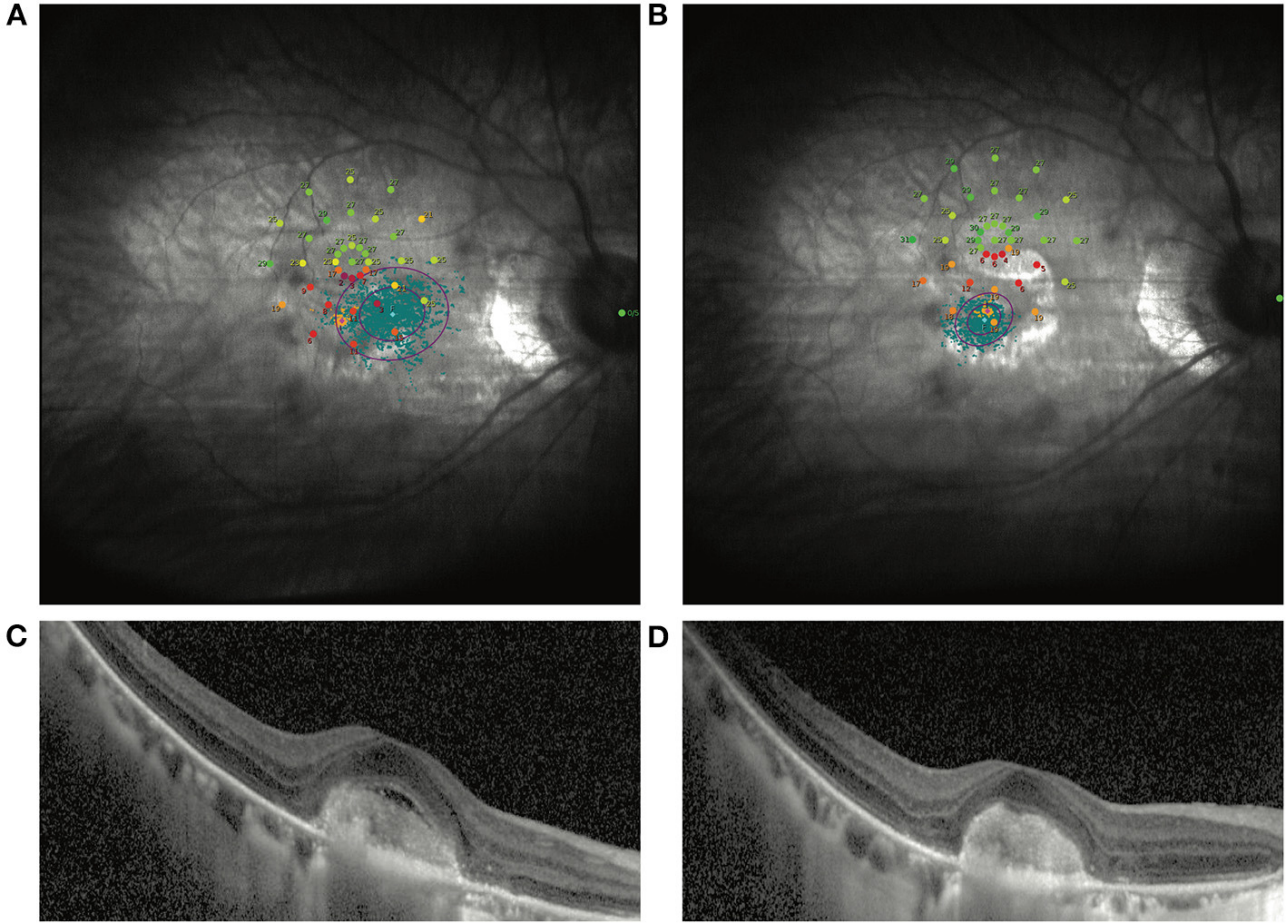
Another representative patient without VA improvement after ranibizumab treatment. **(A)** Shows the image of the MP1 before ranibizumab treatment in an mCNV patient with baseline BCVA as 35 ETDRS letters. After treatment, the VA increased by 3 ETDRS letters. Nevertheless, the macular sensitivity and fixation improved dramatically by MP1 **(B)**. OCT images also show the remission of the CNV lesion in the macular **(C,D)**.

Visual function parameters measured using microperimetry were also compared between groups A and B (Table 4). In group B, the macular integrity did not dramatically change after 3+prn IVR. In the BCVA improved subgroup, the macular integrity improved statistically from 97.86 ± 7.33 (median 100) to 81.95 ± 23.76 (median 96.15) (*p* < 0.0001). However, the average macular threshold in groups A (18.27 ± 5.51 to 22.66 ± 4.93, *p* < 0.0001) and B (18.97 ± 6.99 to 21.93 ± 5.57, *p* = 0.001) both significantly increased. All parameters focusing on the macular fixation stability, including P1, P2, 63% BCEA area, and 95% BCEA area, were all significantly ameliorated no matter the VA improvement or not. No statistical difference could be detected between the two subgroups (Table 4; Figures 1, 2).

Comparing changes of vision function between groups A and B, a board line difference was found only in the change of macular integrity (*p* = 0.06). The improvement of average threshold and retinal fixation were comparable regardless of whether BCVA improved or sustained.

### Multiple Regression Analysis for the Visual Functional Performance After the Treatment

According to the reported possible prognostic factors, age, sex, baseline BCVA, final macular integrity, final average threshold, final P1, final P2, final 63% BCEA area, final 95% BCEA area, final average metamorphopsia score, and final composite VFQ-25 score were included in the multiple linear regression analysis to identify the possible factors that might have influenced the final BCVA. Among these factors, the final BCVA after anti-VEGF treatment correlated only with baseline BCVA (*R* = 0.789, *p* < 0.0001) (Table 6).

**Table 6:** Multiple linear regression model to evaluate the factors associated with the final BCVA.

**Characters**	**Beta**	***P***
Baseline BCVA	0.789	***<0.0001***
Gender		0.403
Age		0.966
Macular integrity		0.317
Average threshold		0.058
P1		0.111
P2		0.131
63% BCEA area		0.160
95% BCEA area		0.205
Average metamorphopsia score		0.159
Composite VFQ-25 score		0.968

*R = 0.789, p < 0.0001*.

We then determined which parameter correlated with the final composite VFQ-25 score. The baseline composite VFQ-25 score and macular integrity were both significantly correlated with the final composite VFQ-25 score, with the betas equal to 0.666 and −0.335, respectively, in a model including gender, age, baseline VFQ-25 composite score, final BCVA, final macular integrity, final average threshold, final P1, final P2, final 63% BCEA area, final 95% BCEA area, and final average metamorphopsia score (*R* = 0.830, *p* < 0.0001) (Table 7).

**Table 7:** Multiple linear regression model to evaluate the factors associated with the final composite VFQ-25 score.

**Characters**	**Beta**	***P***
Baseline composite VFQ-25 score	0.666	***<0.0001***
Macular integrity	−0.335	***<0.0001***
Gender		0.433
Age		0.960
Average threshold		0.308
P1		0.834
P2		0.766
63% BCEA area		0.465
95% BCEA area		0.654
Average metamorphopsia score		0.092
Composite VFQ-25 score		0.721

*R = 0.830, p < 0.0001*.

The change of average metamorphopsia score was the only factor that correlated with the change of the composite VFQ-25 score, with beta = −0.284 and *p* = 0.039, respectively, in a model including gender, age, the change of macular integrity, the change of average threshold, the change of P1, the change of P2, the change of 63% BCEA area, the change of 95% BCEA area, and the change of BCVA (*R* = 0.284, *p* = 0.039) (Table 8).

**Table 8:** Multiple linear regression model to evaluate the factors associated with the change of VR-QoL score.

**Characters**	**beta**	***P***
Gender		0.913
Age		0.091
Change of macular integrity		0.057
Change of average threshold		0.679
Change of P1		0.808
Change of P2		0.338
Change of 63%BCEA area		0.524
Change of 95%BCEA area		0.469
Change of average metamorphopsia score	−0.284	***0.039***
Change of BCVA		0.962

*R = 0.284, p = 0.039*.

## Discussion

In the present study, patients with mCNV underwent a wide range of visual function tests that included microperimetry, m-Charts, VA, as well as subjective judgment of the VR-QoL by VFQ-25 before and after 3+prn anti-VEGF treatments for 1 year. Based on the findings of this retrospective study in mCNV, comprehensive visual function measured by fixation ability, whole macular sensitivity, macular integrity, and the metamorphopsia improved dramatically after anti-VEGF treatment, suggesting the integral lifting in several aspects of visual function. Interestingly, the promotion could still be observed in macular average sensitivity, fixation ability, the metamorphopsia score, and the VFQ-25 score in patients with BCVA improvement ≤ 5 ETDRS letters. That is, even when VA did not improve dramatically after anti-VEGF treatment, these patients nevertheless benefited from anti-VEGF treatment.

VA, especially BCVA in distance, has been considered the gold standard for the assessment of visual function in macular disease, including AMD and mCNV (7, 8, 21). Nevertheless, BCVA only represents the acuity of macular fovea. Surprisingly, as the most commonly used measurement, distance visual acuity failed to correlate with patient-reported visual impairment scores in several diseases as well as in our current study (11–13). However, the metamorphopsia score (22) and general retinal sensitivity (23) correlated with patient-reported VR-QoL in wAMD patients or patients with retinitis pigmentosa. In the present study, we showed that the VR-QoL VFQ-25 score after anti-VEGF treatment was only associated with the baseline VFQ-25 score and macular integrity. Improvements in the VFQ-25 score were associated with changes in the metamorphopsia score. By contrast, no correlation was observed between the BCVA and the VR-QoL.

Microperimetry and m-Charts were widely used in the macular function assessment in AMD and other ocular disease but were barely reported in mCNV (11, 24). The visual function development in mCNV patients with limited BCVA changes was also ignored previously. VA is an important indicator, reflecting part of the visual function. On the other hand, microperimetry and m-Charts are distinct and irreplaceable for quantitative measurement of metamorphopsia, fixation, and general macular sensitivity. As the inexpensive and quickly conducted examination, microperimetry and m-Charts could be examined to obtain a comprehensive understanding of visual function especially in patients with limited or no VA improvement after treatment.

Limitations of this study included the retrospective nature and the relatively limited sample size, possibly explaining the negative results of the change of the VFQ-25 composite score before and after treatment. Twenty patients in the study were also included in a prospective single-blind clinical trial, the SMILE study (NCT03042871). Similar including criteria were used in these two studies to limit the selection bias. Additionally, economic burden may limit the practical application of the multiple examination of visual function in the real world. Furthermore, only 1-year results after the anti-VEGF treatment were analyzed in this study. Future studies with longer follow-up would be greatly beneficial to determining the long-term visual functional improvement.

In conclusion, VA is not the only part that should be noticed following the treatment of mCNV. The severity of macular integrity, macular sensitivity, fixation, and metamorphopsia should also be paid attention after anti-VEGF treatment, especially in patients with limited BCVA gain, whose visual function could still improve. Macular integrity and metamorphopsia, but not VA, were associated with reported VR-QoL, suggesting that VA is far from sufficient for the assessment of visual function. Microperimetry and m-Charts serve as important supplements to measure mCNV treatment responses.

## Data Availability Statement

The raw data are available from the corresponding author by request.

## Ethics Statement

The studies involving human participants were reviewed and approved by Institutional Review Board of Zhongshan Ophthalmic Center, Sun Yat-Sen University. The patients/participants provided their written informed consent to participate in this study.

## Author Contributions

XD supervised the project. SL and XD developed the original idea and wrote the manuscript. SL and LS conducted the statistical analysis. SL, XZ, ZZ, and XL collected the data. All authors contributed to the article and approved the submitted version.

## Conflict of Interest

The authors declare that the research was conducted in the absence of any commercial or financial relationships that could be construed as a potential conflict of interest.

## Publisher's Note

All claims expressed in this article are solely those of the authors and do not necessarily represent those of their affiliated organizations, or those of the publisher, the editors and the reviewers. Any product that may be evaluated in this article, or claim that may be made by its manufacturer, is not guaranteed or endorsed by the publisher.
